# Mapping of long-term cognitive and motor deficits in pediatric cerebellar brain tumor survivors into a cerebellar white matter atlas

**DOI:** 10.1007/s00381-021-05244-2

**Published:** 2021-08-05

**Authors:** Frederik Grosse, Stefan Mark Rueckriegel, Ulrich-Wilhelm Thomale, Pablo Hernáiz Driever

**Affiliations:** 1 Charité-Universitätsmedizin Berlin, Corporate member of Freie Universität Berlin, Humboldt-Universität zu Berlin, and Berlin Institute of Health, Department of Pediatric Oncology and Hematology, Berlin, Germany; 2 Department of Neurosurgery, Universitätsklinikum Würzburg, Würzburg, Germany; 3 Charité-Universitätsmedizin Berlin, Corporate member of Freie Universität Berlin, Humboldt-Universität zu Berlin, and Berlin Institute of Health, Department of Pediatric Neurosurgery, Berlin, Germany

**Keywords:** VLSM, Lesion symptom mapping, Brain tumor, Childhood, Adolescence, Cognitive function, Executive function

## Abstract

**Purpose:**

Diaschisis of cerebrocerebellar loops contributes to cognitive and motor deficits in pediatric cerebellar brain tumor survivors. We used a cerebellar white matter atlas and hypothesized that lesion symptom mapping may reveal the critical lesions of cerebellar tracts.

**Methods:**

We examined 31 long-term survivors of pediatric posterior fossa tumors (13 pilocytic astrocytoma, 18 medulloblastoma). Patients underwent neuronal imaging, examination for ataxia, fine motor and cognitive function, planning abilities, and executive function. Individual consolidated cerebellar lesions were drawn manually onto patients’ individual MRI and normalized into Montreal Neurologic Institute (MNI) space for further analysis with voxel-based lesion symptom mapping.

**Results:**

Lesion symptom mapping linked deficits of motor function to the superior cerebellar peduncle (SCP), deep cerebellar nuclei (interposed nucleus (IN), fastigial nucleus (FN), ventromedial dentate nucleus (DN)), and inferior vermis (VIIIa, VIIIb, IX, X). Statistical maps of deficits of intelligence and executive function mapped with minor variations to the same cerebellar structures.

**Conclusion:**

We identified lesions to the SCP next to deep cerebellar nuclei as critical for limiting both motor and cognitive function in pediatric cerebellar tumor survivors. Future strategies safeguarding motor and cognitive function will have to identify patients preoperatively at risk for damage to these critical structures and adapt multimodal therapeutic options accordingly.

## Introduction

Almost half of all brain tumors in childhood are located in the infratentorial area and nearly a third arises in the cerebellum [1]. Pilocytic astrocytoma (PA) and medulloblastoma (MB) are the most frequent [1], and gross total resection contributes decisively to relapse-free survival [2, 3]. MB patients receive in addition chemotherapy and irradiation [3]. Higher rates of gross total resection and improved adjuvant treatment increased survival rates of MB patients. Thus, long-term deficits, i.e., cognitive, executive, and behavioral abnormalities next to cerebellar symptoms, have come into focus [4–8]. In about 11–29% of patients, these deficits are preceded by a post-operative cerebellar mutism syndrome with key features of muteness and irritability arising within days following surgery [9]. These may be accompanied by cerebellar motor deficits, long-tract signs, and cranial nerve deficits. Long-term sequelae in cerebellar tumor survivors correlate with incidence and extent of post-operative cerebellar mutism syndrome [10]. Schmahmann et al. described a cerebellar cognitive affective syndrome (CCAS) comprising impairments in executive function, visual-spatial cognition, linguistic abilities, and affect regulation in adults [11], and survivors following posterior fossa tumor surgery of pediatric tumors [12]. Postoperative cerebellar mutism syndrome and CCAS are associated with damage to structures composing the Guillain-Mollaret-triangle [13, 14] as well as diaschisis of cerebello-cerebral loops, which were described in animal studies [15] and confirmed in fMRI studies in humans [12, 16, 17]. The corresponding cerebellar topographic organization includes areas for motor tasks in the anterior lobe and lobule VIII, and cognitive tasks (language, spatial cognition, working memory) in the posterior lobe [17]. Even the dentate nucleus (DN) can be divided in a rostrodorsal motor domain and a ventrocaudal non-motor domain [18, 19]. The extent of lesion to the DN and the cerebello-thalamo-cerebral outflow tracts in the superior cerebellar peduncle (SCP) seem to be critical for pattern and severity of long-term deficits [20, 21].

Lesion symptom mapping has been used to map cerebellar function [22]. Studies in pediatric posterior fossa tumor survivors underlined the importance of intact deep cerebellar nuclei and inferior vermis to maintain balance and fine motor function [23–26] as well as undamaged ventral DN, lobules V, VI, VIII, IX, and Crus I for working memory [27]. Albazron et al. found that a postoperative CCAS history mapped to lesions of the cerebellar outflow tract (deep cerebellar nuclei (fastigial nucleus (FN), interposed nucleus (IN), medial DN), superior cerebellar peduncle (SCP), and inferior vermis (IX, VIIIa, VIIIb, X)) in 195 survivors of pediatric cerebellar tumors [28]. We used lesion symptom mapping to correlate long-term motor and cognitive impairment of pediatric cerebellar tumor survivors with consolidated lesions to cerebellar white matter tracts and remaining cerebellar structures. We hypothesized that mapping associates the white matter of the superior cerebellar peduncle (SCP) besides the DN and the posterior lobe with long-term cognitive and motor deficits.

## Patients and methods

### Patients

Cerebellar tumor survivors (Table 1) from our surveillance clinic that non-consecutively underwent surgery between 1990 and 2009 (PA survivors between 1997 and 2009, MB survivors between 1990 and 2007) were approached by mail and more than 2 out of 3 patients that were eligible participated. The local ethics committee approved our retrospective cross-sectional study (EA2/099/06). PA patients had undergone tumor resection only. MB patients underwent adjuvant therapy consisting of craniospinal irradiation with a boost to the posterior fossa and chemotherapy according to the treatment protocols approved by the German Society of Pediatric Oncology and Hematology (GPOH) with comparable levels of neurotoxicity. Three MB patients had single tumor cells in the cerebrospinal fluid at diagnosis, whereas patients with macroscopic metastasis and or brainstem invasion were excluded. Patients and/or a legal guardian gave informed consent. We cannot exclude a bias in our results as patients more strongly affected by long-term motor and cognitive deficits may have been more willing to participate in our study.

**Table 1:** Pediatric cerebellar tumor survivors

	PA	MB
Number	13	18
Sex		
Female	6	9
Male	7	9
Substitution of hormones	-	11
Age at surgery (years)	8.5 (5.4)	8.8 (3.9)
Age at end of therapy (years)	8.8 (4.8)	10.0 (3.8)
Time interval since end of therapy (years)	5.2 (5.8)	6.9 (4.5)
Age at examination (years)	17.2 (9.7)	17.4 (6.3)
Laterality index	0.90 (0.32)	0.80 (0.55)
Leukencephalopathy	0	14
Ventricle drainage at diagnosis	10	16
Hydrocephalus at diagnosis	9	9
Rickham reservoir at examination	0	3
VP-Shunt at examination	0	1

Total numbers and median values with interquartile ranges (IQR)

*PA* pilocytic astrocytoma, *MB* medulloblastoma

### Motor assessment

We assessed ataxia using the International Cooperative Ataxia Rating Scale (ICARS) and corrected for age dependency up to 12 years applying z-scores [29]. The Edinburgh Handedness Inventory identified patients’ handedness [30]. Patients reported weekly practicing time of fine and gross motor function. Fine motor hand movement was assessed using two tasks of different complexity levels on a digital tablet (drawing circles and writing a sentence). We analyzed the kinematic parameters speed and automation as reported elsewhere [7]. The current understanding of the spectrum of postoperative cerebellar mutism includes a broad spectrum of symptoms and clinical signs each modified by post-surgical interval of appearance after surgery, intensity, and duration [9]. Thus, cerebellar mutism as outcome parameter was not addressed in this study as retrospective review of the medical records would have underreported cerebellar mutism.

### Cognitive assessment

Patients underwent HAWIK III (German version of WISC-III for subjects 6–16 years) or HAWIE (German version of WISC-III for subjects older than 16 years) for intelligence estimation. We used the German version of Tower of London (ToL) to explore capability to solve a given problem by mental planning [31], as described previously [32]. Total number of solved problems was compared to an age adjusted comparator group (age corrected percentile rank score (acPRS)) [31]. acPRS and planning time of survivors was compared with results of 41 healthy peers [32]. We selected 3 tasks with stepwise increasing levels of complexity of the Amsterdam Neuropsychological Task (ANT) program for executive function testing: Baseline speed, feature identification, and shifting attention as described elsewhere [32, 33].

### Imaging

MRI examinations were performed during routine surveillance examinations. Imaging was performed with a 3-T MRI system equipped with an eight-channel head coil (Signa Excite; GE Healthcare, Milwaukee, WI) and included a 3D T1-weighted magnetization prepared rapid acquisition gradient echo sequence (MPRAGE) (FOV = 256 mm, number of partitions = 156, voxel size = 1 × 0.86 × 0.86 mm^3^, TR/TE 7872/3248 ms, flip angle 20°) and additional T1- and T2-weighted sequences as described previously [34].

### Voxel-based lesion symptom mapping

Individual cerebellar lesions were drawn manually onto patients’ latest MRI in axial, sagittal, and coronal slices using MRIcron software as volume of interest (VOI). MRI data and VOI were simultaneously normalized into a standard Montreal Neurological Institute (MNI) space using SPM 8 (https://www.fil.ion.ucl.ac.uk/spm/). Normalized VOIs were manually adjusted for introduced spatial errors during normalization. Based on the horizontal (x), sagittal (y), and vertical (z) coordinates of individual normalized cerebellar lesions, the corresponding affected cerebellar structures were defined using the probabilistic atlases of the cerebellar cortex [35], cerebellar nuclei [36], and cerebellar white matter [37]. Only cerebellar structures with at least 5% or more than one-hundred damaged voxels were considered affected. For superimposition of individual stereotaxically normalized cerebellar lesions, right-sided lesions were flipped to the left. For further analysis, patients were grouped for each variable into “impaired” (z-score ≥ 2) and “non-impaired” (z-score < 2). Alternatively, a score differing more than two standard deviations from the mean of healthy age matched peers was used as cut-off. Voxel-based lesion symptom mapping (VLSM) was performed using NPM software. Only voxels damaged in at least 10% of patients were considered for further analysis. For each lesioned voxel, NPM grouped all VOIs in “impaired” and “non-impaired” according to the chosen test variables, and calculated statistical maps with z-scores derived from Liebermeister test for each lesioned voxel. In this study, all voxels having a false discovery rate (FDR) of 0.05 (e.g., 20 real activations for one false positive) are displayed in VLSM images. If VLSM yielded no result, we used subtraction analysis as described elsewhere [25].

### Statistical analysis

For statistical analysis, we used IBM SPSS Statistics Version 26. Due to small sample size, non-parametric distribution of data was assumed and continuous data were described by median and interquartile ranges (IQR). All variables were compared for statistical significance according to tumor histology and lesion location using Mann–Whitney-U test. For VLSM, we analyzed both groups together. Analysis of only PA patients was not possible due to low total number and low total number of survivors with significant deficits.

## Results

### Motor function

Six PA patients and seventeen MB patients had an impaired z-score for ICARS, i.e., above 2 SD. More than half of all MB patients but only one PA patient showed impaired fine motor hand function (frequency (F) and automation (NCV)) in the different tasks. Gross and fine motor practice or time using a computer during leisure time did not differ between groups (Table 2).

**Table 2:** Results of neurological and functional testing according to histology and affected lesion location

		PA (n = 13)	MB (n = 18)	DN + (n = 15)	DN − (n = 16)	SCP + (n = 16)	SCP − (n = 15)	SCP + or DN + (n = 21)	SCP − and DN − (n = 10)
Ataxia	ICARS total score	4 (3)*	9 (13.5)*	11 (16)	6 (5.3)	13.5 (5.3)**	4 (3)**	9.0 (14)	4.0 (3.3)
	ICARS mean z-score	1.4**	9.7**	13.8	5.8	17.2**	2.4**	9.8	3.2
Fine motor hand function	Drawing								
	F	−0.72**	−1.61**	−1.36	−1.10	−1.9	−0.8	−1.3	−1.5
	NCV	0.72**	2.52**	1.89	1.61	2.8*	1.1*	1.7	1.5
	Writing sentence								
	F	−0.6	−1.05	−1.41*	−0.35*	−1.1*	−0.3*	−1.0*	−0.1*
	NCV	0.34	1.22	1.54*	0.22*	1.0**	−0.2**	0.8*	−0.2*
Intelligence	FSIQ	102 (21)	88 (22.5)	88 (14)	102 (24)	87.5 (33)*	102 (25)*	88 (23.5)*	103.5 (16.8)*
	VIQ	100 (19)	93 (14.3)	89 (25)	100 (18)	89 (27)	100 (20)	89 (21.5)*	102 (13.3)*
	PIQ	94 (27)	86.5 (18.5)	84 (9)*	101 (26.3)*	83 (15.8)*	97 (27)*	84 (13)**	105.5 (19.5)**
Planning	ToL								
	acPRS	24 (56)	38.5 (51.3)	24 (81)	49.5 (51.3)	28.5 (63)	53 (67)	24 (64.5)	63 (44)
Executive function	Baseline speed	−1.16**	−0.03**	−0.47	−0.89	0.0**	−1.0**	−0.4	−0.9
	Feature identification								
	Dissimilar	−0.77	−0.11	0.06	−0.46	0.2	−0.7	−0.2	−0.5
	Similar	0.80**	0.92**	0.16	−0.07	0.2	−0.2	0.1	−0.2
	Shifting attention								
	Compatible fixed	−0.27**	1.84**	1.05	0.48	1.6*	0.2*	1.1	0.3
	Incompatible fixed	1.05	0.90	1.48	0.71	1.1	0.6	1.3	0.5
	Compatible random	0.63	1.34	1.02	0.97	1.4	0.9	1.3	0.4
	Incompatible random	0.28*	1.28*	0.90	0.59	1.3*	0.3*	1.2	0.2

Median values and interquartile ranges (IQR)

*PA* pilocytic astrocytoma, *MB* medulloblastoma, *DN* + lesion in dentate nucleus *DN − *no lesion in dentate nucleus, *SCP* + lesion in superior cerebellar peduncle, *SCP − *no lesion in superior cerebellar peduncle, *F* frequency*, NCV* automation of fine motor hand function, *FSIQ* full scale intelligence quotient, *VIQ* verbal IQ, *PIQ* performance IQ, *ToL* Tower of London*, acPRS* age-corrected percentile rank score

^*^p < 0.05, **p < 0.01

### Cognitive function

Full-scale IQ (FSIQ), performance IQ (PIQ), and verbal IQ (VIQ) were significantly impaired in 8 (2 PA, 6 MB), 12 (5 PA, 7 MB), and 6 (2 PA, 4 MB) survivors, respectively. Planning capability was significantly compromised in PA (24%) and MB (39%) patients when compared to healthy peers (PA: p < 0.001, MB: p < 0.001) including prolonged planning time with increasing task complexity (PA: p = 0.025, MB: p < 0.001). PA patients showed delayed response time in executive function only at increased task complexity. Response time of MB patients was more frequently delayed (z-score > 2) with significant difference compared to PA patients. When focusing on committed errors (z-score > 2), PA and MB patients committed more errors with increasing task complexity but without significant difference according to tumor entity. Thus, response time was prolonged in MB patients but not decision quality in terms of number of errors (Table 2). Hydrocephalus at diagnosis in both survivor groups did neither influence motor nor cognitive function.

### Lesion characteristics

**Fig. 1:**
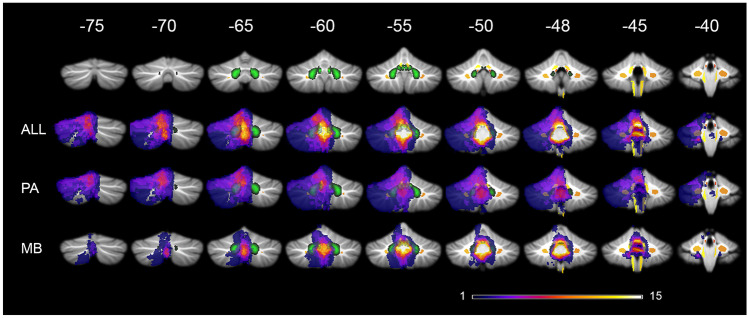
Lesion overlay in pediatric cerebellar tumor survivors. Legend to Fig. 1: PA pilocytic astrocytoma, MB medulloblastoma. Color code indicates numbers of overlapping lesions. Arabic numbers indicate y-coordinates in MNI space. Right-sided lesions being flipped to the left on cerebellar template with deep cerebellar nuclei in green and white matter tracts in yellow/orange

Maximum overlap for all lesions of cerebellar tumor survivors was seen in inferior vermis; paravermal lobules IX, VIIIa, VIIIb, VI, and V; inferior medial lobule IV; the deep cerebellar nuclei; and the SCP close to its origin at the ventrocaudal DN as well as the inferior cerebellar peduncle (ICP) close to inferior lobule IV and the SCP (Fig. 1; Table 3). Lesions of MB tumor survivors showed highest overlap in inferior vermis, paravermal lobule IX, the SCP, the ICP, and the deep cerebellar nuclei. In PA tumor survivors, highest lesion overlap was located more cranial comprising the vermis; paravermal lobule IX, V, and VI; dorsomedial parts of CRUS I and II, and the deep cerebellar nuclei, while the ICP, SCP, and cerebellar hemispheres were less often affected (Fig. 1; Table 3).

**Table 3:** Individual cerebellar lesions in pediatric cerebellar tumor survivors

Pat	Histo	White matter tracts	Nuclei	Vermal (x ≤ ± 10)	Hemisphere	
					Paravermal (− 24 ≤ x ≤ − 10) (+ 10 ≤ x ≤ + 24)	Lateral (x < − 24, x > + 24)
1	PA	-	R: DN		R: VI, Crus I + II	R: VI, Crus I + II
2	PA	L: SCP, ICP R: SCP, ICP	L: DN, IN, FN R: IN, FN	I IV, V, VI, VIIb, VIIIa + b, IX, X, Crus I + II	L: V, VI, VIIb, VIIIa, IX, Crus I + II R: IX	L: Crus I
3	PA	R: SCP, ICP	R: IN, FN L: IN, FN	I IV, V, VI, VIIIa + b, IX, X	R: V, VI	
4	PA	R: SCP, ICP	R: DN, IN, FN L: FN	I IV, V, VI, VIIb, VIIIa + b, IX, X, Crus I + II	R: I IV, V, VI, VIIb, VIIIa + b, IX, X, Crus I + II	R: V, VI, VIIb, VIIIa + b, X, Crus I + II
5	PA	R: SCP, ICP, MCP L: SCP	R: DN, IN, FN L: DN, IN, FN	I IV, V, VI, VIIb, VIIIa + b, IX, X	R: I IV, V, VI, VIIb, VIIIa + b, IX, X Crus I + II L: IX	R: V, VI, VIIb, VIIIa + b, Crus I + II
6	PA	-		I IV		
7	PA	R: ICP		IX, X		
8	PA	-	L: DN, IN	V, VI, VIIb, VIIIa, Crus I + II	L: VI, Crus I + II	L: Crus I
9	PA	-	L: DN, IN	V, VI, VIIb, VIIIa, Crus I + II	L: VI, Crus I + II R: VIIb, Crus II	
10	PA	-	L: IN, FN R: IN, FN	V, VI, VIIb, VIIIa, Crus I + II	L: VI	
11	PA	R: MCP	R: DN		R: I IV, V, VI, Crus I + II	R: V, VI
12	PA	L: ICP R: ICP	R: IN, FN L: IN, FN	I IV, V, VI, VIIb, VIIIa + b, IX, X, Crus II	R: IX	
13	PA	-	L: IN	V, VI, VIIb, VIIIa, Crus I + II	L: V, VI	
14	MB	R: SCP, ICP L: ICP	L: IN, FN R: IN, FN	I IV, V, VI, VIIb, VIIIa + b, IX, X, Crus II	R: IX	
15	MB	R: SCP	L: DN, IN, FN R: DN, IN, FN	VIIb, VIIIa + b, IX, X	R: IX	
16	MB	R: SCP, ICP L: ICP	L: IN, FN R: DN, IN, FN	I IV, V, VI, VIIb, VIIIa + b, IX, X	L: IX R: IX	
17	MB	R: SCP	L: IN, FN R: IN, FN	I IV, VIIIb, IX, X	R: IX, X	
18	MB	-	L: FN R: IN, FN	VIIb, VIIIa + b, IX, X, Crus II		
19	MB		L: FN R: IN, FN	VIIIa + b, IX, X	R: IX	
20	MB	R: SCP	L: FN R: IN, FN	IX, X	L: IX R: IX	
21	MB	R: SCP, ICP	L: FN R: DN, IN, FN	I IV, VIIb, VIIIa + b, IX, X	R: VIIb, VIIIa + b, IX, Crus II	R: VIIb
22	MB	R: ICP	L: FN R: DN, IN, FN	I IV, V, VI, VIIb, VIIIa + b, Crus I + II	R: V, VI	
23	MB	-	L: IN, FN R: IN, FN	IX, X	R: IX	
24	MB	R: SCP		VIIb, VIIIa + b, IX, X, Crus II		
25	MB	R: SCP, ICP L: SCP, ICP	L: DN, IN, FN R: IN, FN	I IV, V, VI, VIIb, VIIIa + b, IX, X, Crus II	L: VIIIb, IX, X R: IX, X	
26	MB	-		IX, X	L: IX	
27	MB	R: SCP, ICP L: SCP	L: IN, FN R: IN, FN	VIIIa + b, IX, X	L: IX R: IX	
28	MB	R: SCP, ICP L: SCP, ICP	L: DN, IN, FN R: IN, FN	I IV, VIIb, VIIIa + b, IX, X	L: IX R: IX	
29	MB	-		I IV, V, VI	L: V, VI	
30	MB	R: SCP, ICP L: SCP, ICP	L: DN, IN, FN R: DN, IN, FN	I IV, V, VI, VIIb, VIIIa + b, IX, X	L: IX, X R: I IV, V, VIIIb, IX	
31	MB	R: SCP, ICP L: SCP	L: IN, FN R: DN, IN, FN	VIIIa + b, IX, X	L: IX R: IX	

Latin numbers denote affected cerebellar lobules. Arabic numbers denote coordinates in SUIT template

*Pat* patient, *PA* pilocytic astrocytoma, *MB* medulloblastoma, *SCP* superior cerebellar peduncle, *ICP* inferior cerebellar peduncle, *MCP* median cerebellar peduncle, *R* right, *L* left, *DN* dentate nucleus, *IN* interposed nucleus, *FN* fastigial nucleus

### Association of lesion with function

#### White matter tracts

The SCP was lesioned in 16 cerebellar tumor survivors, while the ICP was affected at its cerebellar segment next to the SCP and inferior lobule IV in 15 tumor survivors. Survivors with lesion to the SCP showed significantly pronounced ataxia, impaired fine motor hand function in writing, impaired cognitive function, and impaired executive function (Table 2), while lesion to the ICP only was significantly associated with worsened ataxia (ICARS total score: p = 0.021, z-score for ICARS: p = 0.004).

#### Dentate nucleus

Fifteen cerebellar tumor survivors had a persisting lesion within the DN. They exhibited significantly impaired fine motor function and PIQ (Table 2).

### Voxel based lesion symptom mapping

#### Motor function

**Fig. 2:**
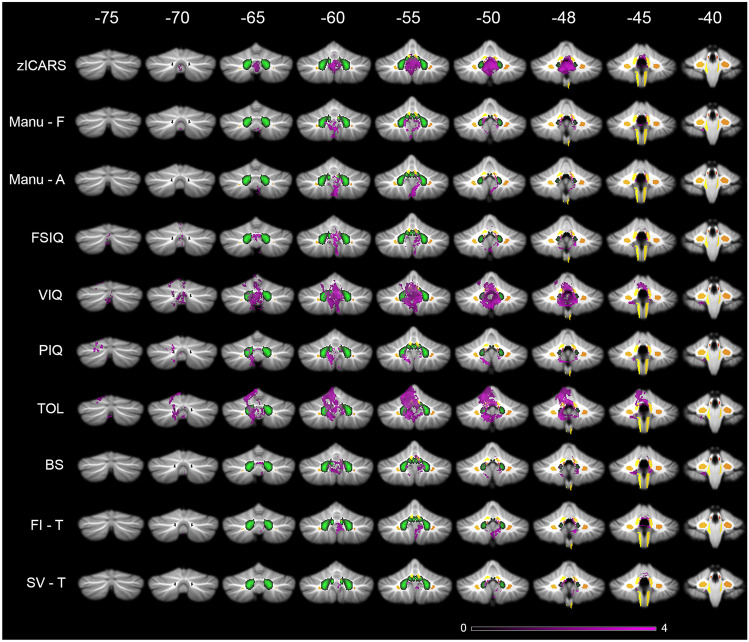
Lesion symptom maps of motor and cognitive function in pediatric cerebellar tumor survivors. Legend to Fig. 2: zICARS z-score of total ICARS score, Manu-F frequency of fine motor hand movement, Manu-A automation of fine motor hand movement, FSIQ full scale intelligent quotient, VIQ verbal IQ, PIQ performance IQ, TOL age corrected percentile rank score of Tower of London, BS baseline speed (ANT), FI-T reaction time at feature identification among similar patterns (ANT), SV-T reaction time of shifting attention at attention flexibility (ANT). Deep cerebellar nuclei are shown in green, white matter tracts in yellow/orange. Color code indicates z-score in lesion symptom map. Arabic numbers indicate y-coordinates in MNI space

Lesion maps of survivors with higher ICARS z-scores comprised the proximal superior cerebellar peduncle (SCP) near its origin in the ventrocaudal DN, the vermis, IN, FN, and small parts of inferior medial lobule IV (Fig. 2). Deficits in frequency or automation of fine motor hand function likewise mapped to the SCP, inferior anterior vermis, IN, FN, and in addition to ventrocaudal-medial DN and parts of the ICP in the superior vermal area, where it is located next to the SCP and inferior lobule IV (Fig. 2).

#### Intelligence

Significant deficits in FSIQ mapped to the proximal SCP, the vermis, IN, and FN (Fig. 2). Lesion maps of PIQ depicted predominantly paravermal areas (lobule VI, Crus I, VIIIa, VIIIb), inferior vermal lobule IX, IN, dorsal and medial DN, and a possible association of the proximal SCP close to the ventrocaudal DN. For VIQ, lesion maps included the SCP, an intense vermal distribution, IN, FN, and small parts of the ICP. In addition, paravermal area of lobule V, VI, Crus I, and dorsal and medial parts of DN were mapped (Fig. 2).

#### Planning and executive function

**Fig. 3:**
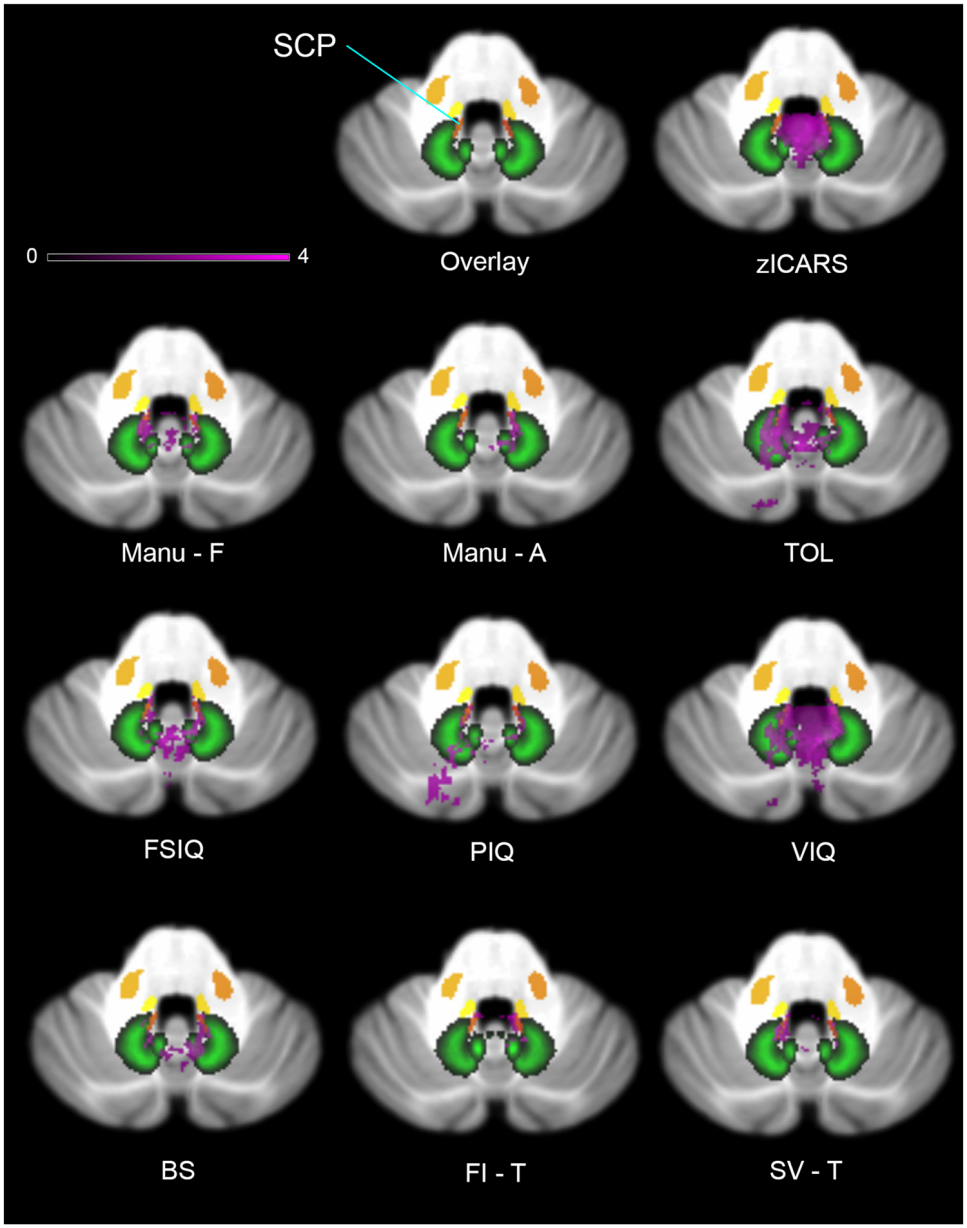
Involvement of proximal SCP in lesion symptom maps of motor and cognitive function in pediatric cerebellar tumor survivors. Legend to Fig. 3: zICARS z-score of total ICARS score, Manu-F frequency of fine motor hand movement, Manu-A automation of fine motor hand movement, FSIQ full scale intelligent quotient, VIQ verbal IQ, PIQ performance IQ, TOL age corrected percentile rank score of Tower of London, BS baseline speed (ANT), FI-T reaction time at feature identification among similar patterns (ANT), SV-T reaction time of shifting attention at attention flexibility (ANT). Deep cerebellar nuclei are shown in green, white matter tracts in yellow/orange. Color code indicates z-score in lesion symptom map. Slices show lesion symptom maps at coordinate z =  − 32 in MNI space

Significant deficits in planning (acPRS) mapped to the SCP, vermis, IN, FN, medial DN, paravermal lobules (V, VI, Crus I + II, VIIb, VIIIb, IX), and small part of the ICP in the superior vermal area, where it is located next to the SCP and inferior lobule IV (Fig. 3). Statistical maps for relevant deficits in feature identification (working memory) comprised the SCP, the inferior vermis, and inferior medial lobule IV (Fig. 2). Lesion maps for the shifting attention task included the SCP, inferior vermis, ventromedial DN, and inferior medial lobule IV.

## Discussion

Using the atlas of cerebellar white matter tracts [37] for VLSM of long-term sequelae in pediatric cerebellar tumor survivors, we demonstrated that lesions of the SCP were critical for impaired motor as well as cognitive performance. The SCP at its proximal part close to ventrocaudal DN was involved in every tested motor function (ataxia, frequency, and automation of fine motor hand movement) as well as for deficits of cognition (FSIQ, PIQ, VIQ, feature identification, attention flexibility, and mental planning) (Fig. 3).

Our results are in line with our previous studies employing diffusion tensor imaging (DTI) and tractography to investigate cerebello-cerebral pathways when we found significantly reduced frontocerebellar tract volumes and reduced fractional anisotropy (FA), resembling white matter damage, in frontal white matter and the SCP in pediatric survivors, and a history of postoperative cerebellar mutism [16] as well as impaired long-term cognitive function (FSIQ, attention flexibility) [34]. Law et al. discovered an association of reduced FA and increased diffusivity in cerebello-thalamo-cerebral tracts and impaired working memory in pediatric cerebellar tumor survivors, thereby corroborating our current and previous findings [38]. Gomes et al. reported a significant correlation of reduced FA in SCP and motor learning deficits in 18 long-term survivors of low-grade pediatric posterior fossa tumor and healthy peers [39]. VLSM in 195 long-term survivors after cerebellar pediatric tumor removal associated lesions of the SCP, the deep cerebellar nuclei (FN, IN, medial DN), and inferior vermis (IX, VIIIa, VIIIb, X) to CCAS in the postoperative period as reported by medical records [28]. These studies and our current highlight the critical impact of lesions to the SCP for integrity of motor (balance and fine motor hand) and higher cognitive functions. Concerning the ICP an impact on ataxia and dysmetria in fine motor function may appear plausible as it solely comprises afferent information from the spinal cord. So far, no reports on damage of the ICP following tumor surgery and motor impairment are available. As involvement of ICP in lesion maps only comprises small areas, which were also included in lesion maps of cognitive function, a false positive finding cannot be fully excluded.

VLSM revealed an association of lesions of the DN with motor deficits as well as planning, mental flexibility, PIQ, and VIQ (Figs. 2 and 3). IN and FN were involved in all impaired motor tasks, mental planning, FSIQ, and VIQ. Involvement of the deep cerebellar nuclei in motor and cognitive tasks is in accordance with the current literature [18, 21, 25]. Previous lesion studies on ataxia in pediatric cerebellar tumor survivors reported significant associations between lesions to IN and FN as well as dorsomedial portions of DN [23, 24, 40]. More detailed analysis indicated that FN and IN are predominantly important for balance control [23, 24, 26, 40], whereas IN and DN play an important role for upper and lower limb movement [23, 40]. Küper et al. demonstrated a regain of balance and upper limb function following reduction of edema to DN, whereas persisting impairment was associated with permanent lesion of the deep cerebellar nuclei and vermis [25]. Puget et al. reported a correlation between extent of damage to the DN and motor deficits as well as cognitive impairments (reduced FSIQ) in sixty-one pediatric posterior fossa tumor survivors 5.6 years after multimodal therapy [21]. Our study is the first that showed association of DN to deficits in executive function (mental planning, shifting attention). Involvement of IN and FN to varying extent in lesion maps of cognitive function (ToL, FSIQ, VIQ) may reflect motor deficits as each test comprised motor components. So far, only Albazron et al. found an association of the CCAS following cerebellar tumor removal in pediatric patients and lesion to the cerebellar outflow tract with peak finding in FN and suggested its involvement in higher cognitive functions [28]. Apart from this, no association of FN or IN to cognitive function was reported.

Our findings in VLSM included the inferior vermis (VIIIb, IX) in every tested motor and cognitive function. In the majority of lesion maps, vermal representation comprised lobule VIIIa and especially for ataxia lobule X. Several studies described lesion involvement of the inferior vermis besides the deep cerebellar nuclei as critical risk factor for persisting motor (ataxia, dysmetria, balance, intention tremor) and cognitive impairments (PIQ, CCAS) [21, 25, 28].

### Limitations

An important limitation of our study is the low number of survivors of each patient group (PA, MB) as different biology implies different local damage pattern. Next, chemotherapy, irradiation, and hydrocephalus contribute to general neurotoxicity and may be confounders beyond the local damage to the cerebello-cerebral tracts. Still, adjuvant treatment toxicity seems to play an increasingly inferior role as survivors of medulloblastoma of the Sonic hedgehog Group, who exhibit inferior chances of damage to the SCP and DN due to more lateralized tumor location, are characterized by superior cognitive performance despite comparable general treatment neurotoxicity [41].

## Conclusion

We identified lesions of the SCP in pediatric cerebellar tumor survivors next to DN and the inferior vermis to be associated with impaired motor, cognitive, and executive function. Preventing surgical harm to the SCP and deep cerebellar nuclei seems to be critical for preserving optimal motor and cognitive function in pediatric cerebellar tumor survivors. As gross total tumor resection is critical for lowering event-free survival in both patient groups an approach to safeguarding motor and cognitive function may lead to conflicting aims in the management of these patients. A solution could be using radiologic algorithms of conventional MRI as well as fMRI and DTI that may identify preoperatively patients at risk of developing postoperative cerebellar mutism [42, 43] which is the most predictive complication for long-term deficits [44]. Advanced MRI radiomics [45] and liquid biopsy from cerebrospinal fluid [46] may further enhance preoperative diagnostic accuracy. In case of impending high risk for damage of critical cerebro-cerebellar loops, these methods may open a window for neo-adjuvant antitumoral therapy that could contribute to complete tumor resection without compromising integrity of deep cerebellar nuclei and the SCP [47, 48]. Future studies will have to address gene polymorphisms determining neurodegeneration as well as neuroregeneration [49] next to neuroprotective and neurorehabilitation measures that contribute to the pattern of functional deficits [50].

## Data Availability

Data is available on figshare.com (https://doi.org/10.6084/m9.figshare.13653269).
